# Management of Traumatic Liver and Bile Duct Laceration

**DOI:** 10.5005/jp-journals-10018-1247

**Published:** 2017-09-29

**Authors:** Charu Tiwari, Hemanshi Shah, Mukta Waghmare, Kiran Khedkar, Pankaj Dwivedi

**Affiliations:** 1 Department of Paediatric Surgery, Topiwala National Medical College & BYL Nair Charitable Hospital, Mumbai, Maharashtra, India

**Keywords:** Bile leak, Biloma, Hepatic trauma.

## Abstract

Posttraumatic major bile leak in children is uncommon, with few cases reported in the literature. These injuries are seen in high-grade liver trauma and are difficult to diagnose and manage. We describe a 7-year-old boy with grade IV hepatic trauma and bile leak following blunt abdominal trauma. The leak was successfully managed by percutaneous drainage and endoscopic retrograde cholangiopancreatography (ERCP) stenting of the injured hepatic duct.

**How to cite this article:** Tiwari C, Shah H, Waghmare M, Khedkar K, Dwivedi P. Management of Traumatic Liver and Bile Duct Laceration. Euroasian J Hepato-Gastroenterol 2017;7(2):188-190.

## INTRODUCTION

The incidence of biliary complications after blunt hepatic trauma has been reported to be 2.8 to 7.4%.^1-3^ Such complications are usually seen in high-grade liver injuries and are more complex unlike postoperative iatrogenic injuries.^4^ The management of these injuries requires a high index of clinical suspicion, timely and correct diagnosis, and appropriate treatment.^4,5^ This reduces complications and helps in rapid recovery of the patient.

## CASE SUMMARY

A 7-year-old boy was bought with trauma to head, right upper limb, and abdomen due to a road traffic accident. At admission, he had tachycardia and hypotension. Glasgow Coma Scale score was 15/15. Abdomen was distended with tenderness and guardingin the right hypochondrium. He was resuscitated by administration of crystalloids and colloids.

**Fig. 1:**
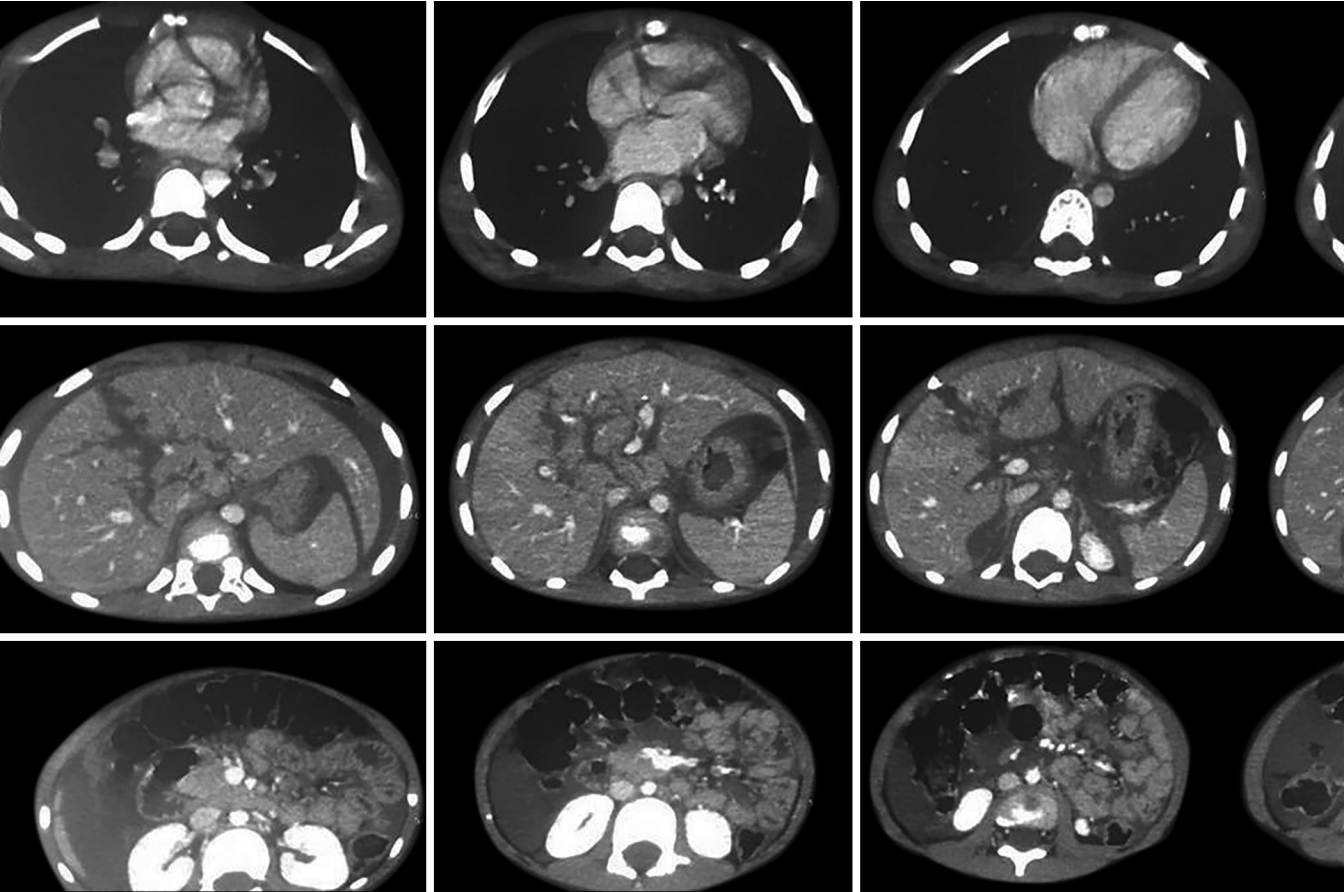
Contrast-enhanced computed tomography of abdomen showing massive hemoperitoneum with AAST grade IV trauma in segments III and IV of liver and AAST grade I trauma in spleen

Abdominal sonography revealed gross hemoperito-neum with liver laceration in segments III and IV along with splenic laceration. Contrast-enhanced computed tomography (CECT) abdomen suggested massive hemo-peritoneum with American Association for the Surgery of Trauma (AAST) grade IV trauma in segments III and IV of liver and AAST grade I trauma in spleen (Fig. 1). Bowel was normal. There was no pneumoperitoneum. The CT thorax was unremarkable. The right humeral fracture was managed with a cast. The patient was kept under strict observation with regular monitoring of vitals. Blood was transfused.

**Figs 2A and B:**
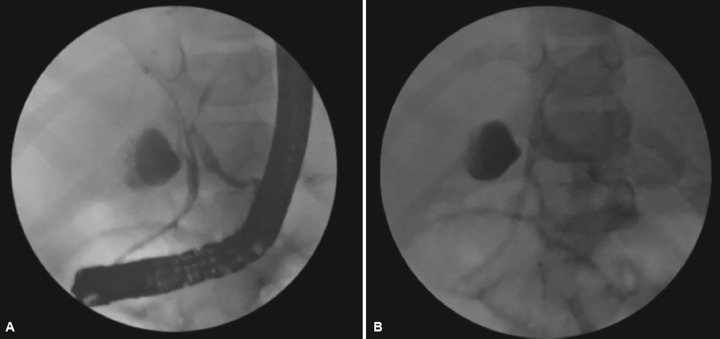
Endoscopic retrograde cholangiopancreatography showing bile leak from left hepatic duct

Patient’s vitals stabilized. He tolerated oral diet and was opening bowel regularly. However, he continued to have persistent abdominal distention for 5 days after the injury. Repeat ultrasonography showed massive fluid collection with moving echoes and septae. Biliary ooze from the lacerated liver edge was suspected. Aspiration confirmed golden yellow bile. Ultrasound-guided pig tail for external drainage was inserted and 1,000 cc bile was drained. Bile drainage continued to be constant at 300 cc per day. The ERCP revealed left hepatic duct injury which was stented with 7 French stent (Fig. 2). External drain was removed after decrease in output. The patient recovered well. Stent was removed after 6 weeks.

## DISCUSSION

Complications are mostly seen in high-grade liver inju-ries.^4,6^ Such complications can be bile leak, liver abscess, and ischemic necrosis of the liver and gallbladder.^4^ Injuries to the biliary tract after blunt abdominal trauma can be classified as intra- or extrahepatic. Extrahepatic bile duct injury may occur in the absence of a liver paren-chymal injury, whereas intrahepatic bile duct injury is invariably associated with liver parenchymal laceration.^4,7^ These injuries can be simple bile leaks into the lacerated liver, peritoneal cavity, or pleural cavity or even biliary-vascular fistulae.^4,8^

The appearance of clinical signs and symptoms like right upper quadrant pain, jaundice, fever, or malena indicates liver-related complications.^1^ Studies in literature show that the optimal time period from injury to appearance of complications in high-grade liver injuries is 7 to 10 days.^1^ A repeat CECT scan has been recommended during this time period in symptomatic patients with high-grade liver trauma.^1^ The CECT suggests biloma as progressive growth of a well-circumscribed, low-attenuation intraparenchymal or perihepatic collection.^1,9^

However, though the presence of free fluid is sensitive, it is nonspecific for bile leak.^4,10^ Delayed bile leaks have also been reported to occur following a secondary rupture of a subcapsular collection or due to duct ischemia.^4,7^ For asymptomatic patients with low-grade liver injuries, a repeat CECT is generally unnecessary.^1,11^

The ERCP with stenting coupled with percutaneous drainage is the mainstay of therapy for the treatment of major bile leaks in patients with high-grade liver trauma.^1^ Endoscopic techniques that are used to manage bile leaks include biliary sphincterotomy alone, biliary stenting with or without sphincterotomy, and nasobiliary drainage with or without sphincterotomy to allow internal drainage of bile and reduction in intrabiliary pressure that would help the bile duct injury to seal off.^4,12^ Wahl et al^13^ have suggested that patients sustaining high-grade hepatic injury were more likely to have bile leaks.^4^ It has been recommended that continuous high-output biliary drainage should be managed by ERCP and stenting to allow for healing.^1^ Marks et al^14^ have suggested that stenting, rather than sphincterotomy was more effective in resolving biliary leaks.^1^ On the contrary, most of the minor peripheral biliary leaks usually seal spontaneously without any intervention.^1^

The success rate for ERCP and stenting ranges from 90 to 100%.^1,15-17^ Sugimoto et al,^18^ Bajaj et al,^19^ and Bala et al^1^ have reported success of ERCP in blunt hepatic trauma-related biliary leaks. Nonoperative treatment of biliary complications is associated with little or no long-term morbidity.
